# Ideas, actors and institutions: lessons from South Australian Health in All Policies on what encourages other sectors’ involvement

**DOI:** 10.1186/s12889-017-4821-7

**Published:** 2017-10-16

**Authors:** Fran Baum, Toni Delany-Crowe, Colin MacDougall, Angela Lawless, Helen van Eyk, Carmel Williams

**Affiliations:** 1 Southgate Institute for Health, Society and Equity, Flinders University, Adelaide, Australia; 2 College of Medicine and Public Health, Flinders University, Adelaide, Australia; 3 College of Nursing and Health Sciences, Flinders University, Adelaide, Australia; 4 Health Determinants and Policy, Department for Health and Ageing, Adelaide, Australia

**Keywords:** Health in All Policies, Institutional theory, Intersectoral collaboration, Social determinants of health

## Abstract

**Background:**

This paper examines the extent to which actors from sectors other than health engaged with the South Australian Health in All Policies (HiAP) initiative, determines why they were prepared to do so and explains the mechanisms by which successful engagement happened. This examination applies theories of policy development and implementation.

**Methods:**

The paper draws on a five year study of the implementation of HiAP comprising document analysis, a log of key events, detailed interviews with 64 policy actors and two surveys of public servants.

**Results:**

The findings are analysed within an institutional policy analysis framework and examine the extent to which ideas, institutional factors and actor agency influenced the willingness of actors from other sectors to work with Health sector staff under the HiAP initiative. In terms of ideas, there was wide acceptance of the role of social determinants in shaping health and the importance of action to promote health in all government agencies. The institutional environment was initially supportive, but support waned over the course of the study when the economy in South Australia became less buoyant and a health minister less supportive of health promotion took office. The existence of a HiAP Unit was very helpful for gaining support from other sectors. A new Public Health Act offered some promise of institutionalising the HiAP approach and ideas. The analysis concludes that a key factor was the operation of a supportive network of public servants who promoted HiAP, including some who were senior and influential.

**Conclusions:**

The South Australian case study demonstrates that despite institutional constraints and shifting political support within the health sector, HiAP gained traction in other sectors. The key factors that encouraged the commitment of others sectors to HiAP were the existence of a supportive, knowledgeable policy network, political support, institutionalisation of the ideas and approach, and balancing of the economic and social goals of government.

## Background

For four decades health promoters have concluded that to be most effective, health promotion strategies must involve government agencies beyond the health sector. Despite this there is little evidence showing what factors encourage other sectors to see health promotion as part of their mission. The cross sectoral mandate has been central to the new public health [1, 2]. The Alma Ata Declaration urged “intersectoral action for health” [3]. The Ottawa Charter argued for “Healthy Public Policy” [4] which Milio defined as ‘multisectoral in scope, and participatory in strategy’ [5].

To achieve healthier societies and communities it is argued that there must be improvements in the coordination of government departments to tackle complex and “wicked” health and social problems in a joined up fashion [6, 7]. More recently Health in All Policies (HiAP) has extended intersectoral action by facilitating sectors outside of Health to routinely consider and account for the health impact of their policies, plans and implementation [8–12]. The approach was adopted in South Australia in 2007 and has been a constant presence in the State’s policy response to the social determinants of health and health equity [13, 14]. Our longitudinal evaluation of this initiative is one of the few attempts to map HiAP implementation and to understand what accounts for success and failure. Although much literature asserts the importance of HiAP there is very little empirical evidence and a particular gap in understanding about how to encourage other sectors to participate in HiAP.

The literature on policy implementation is scant despite its importance to achieving improved societal outcomes, including in health, but does provide a theoretical base for studying intersectoral action. Policy literature no longer sees policy implementation as linear but rather as interactions between context, content, process and power [15]. Exworthy suggests implementation is disjointed and messy [16]. Kickert et al. argue that policy-making takes place in networks that are made up of various actors (including individuals, coalitions and organizations), none of which can determine the other actors’ strategies [17]. Institutional theory is helpful to unpack structure and agency in the process of policy implementation [18–20]. Scott identifies the regulative, normative and cultural-cognitive pillars of institutions, which explain how and why institutions, and the actors therein, operate [20]. Understanding how institutions shape the HiAP activities and how actors react to institutional practices promises to be helpful in making sense of the contexts within which HiAP is operating. Howlett, Ramesh and Perl similarly note that policy problems are engaged and responses are crafted in policy worlds which have a distinctive constellation of actors (people and networks involved), ideas (the content of policy) and institutions (structures, rules and mandates) [18]. Each of these elements interacts and leads to a particular form of policy implementation. Knowledge about how this interaction happens promises to assist health promoters in understanding how other sectors can be persuaded to maximise their beneficial health impact. Exworthy and Powell argue that effective implementation requires clear policy objectives, feasible mechanisms and processes to meet these objectives, and adequate resources to finance them [21]. They also stress the importance of understanding the interdependent context in which governance operates and the need to study policy implementation in the ‘congested state’ [22, 23] which emphasises fragmented and plural forms of governance. The notion of a congested state with a consideration of the key role of institutions in shaping policy, the ideas underpinning policies and plans as they are implemented, and the agency of actors in responding to and shaping institutional constraints promises to be a robust framework to examine the ways in which other sectors engage with HiAP.

This paper examines the extent to which actors from sectors other than health engaged with the South Australian Health in All Policies initiative and determines why they were prepared to do so and the mechanisms by which successful engagement happened. This examination draws on theories of policy development and implementation. We note that the focus of this paper is not on the health and equity outcomes of the SA HiAP initiatives as these will be examined in detail in a paper that is currently being prepared. In this paper we are concerned with a detailed examination of the HiAP process and the factors that encourage other government sectors to become involved.

## Methods

The data were collected over five years (2012–2016) as part of a mixed-methods research project addressing the question ‘Does a Health in All Policies approach improve health, equity and wellbeing?’ This paper draws on documentation of key events and documents, semi-structured interviews and two waves of an electronic survey of public servants. Policy makers appear to value mixed methods in research to inform policy. In our study, qualitative data informed the development of the survey questions which were in turn interpreted, aided by further qualitative data. There were no competing truth claims between the different data sets and we used descriptive statistics on a census, rather than a sample; conforming to elaborative mixed methods whereby each method illustrates, but does not contradict, the other [24, 25].

### Documentation of key events and documents

A log of key policy events and details of HiAP’s engagement with other sectors was maintained over five years. This log documented observations made by research staff during regular visits to government offices and included a timeline of key changes in the political and bureaucratic environments affecting HiAP. Thematic document analysis of HiAP documents (meeting notes, event attendance lists as well project proposals, work summaries and project reports) was undertaken during 2013–2016 using the qualitative analysis software NVivo 11. This analysis revealed details of how HiAP staff engaged with other sectors, how intersectoral engagement changed over time following contextual developments, and highlighted which departments and actors were involved.

### Semi-structured interviews

This paper draws on a subset of the interviews undertaken between January 2013 and June 2016 for the broader HiAP evaluation. This subset involves 112 interviews with 64 individuals. The paper primarily draws on 49 interviews with staff from non-health departments/agencies of the South Australian Government public sector, but also, where data from interviews relate to the involvement of other sectors in HiAP, 54 with staff from the Health Department, five with academics with experience of the HiAP initiative and four with politicians or political staff. The 49 interviews with other sectors involved 33 public servants from departments/agencies other than Health. Ten of these 33 individuals were interviewed multiple times (between two and four times) given their roles in several HiAP projects, or the need to capture developments over time. These interviews involved public servants from 15 State Government departments and agencies who have had contact with the HiAP approach between 2007 and 2016. The 15 departments/agencies included: community services, education, justice, transport, governance, infrastructure, employment, trade and natural resources. The later interview schedules were adapted in light of emerging information from the earlier interviews and the survey so that, for instance, more detailed questions were asked about the impact of the changing economic climate and its impact on the public service.

Interviews were undertaken by six academics experienced in qualitative interviewing. Questioning was guided by pre-prepared interview schedules. The interviews were recorded and transcribed verbatim for all but two respondents. The interviews averaged 40 min, ranging from 15 min to 1 h 35 min. All interviewees were offered the chance to review their transcript and seven chose to check and amend their transcript by either editing the transcript directly or sending email notes to clarify or elaborate on a particular aspect of the interview. Two people asked that the interview not be recorded, but rather written notes be taken by the interviewer. In this case the notes were checked by the interviewee.

#### Analysis of interviews

A collaborative, thematic analysis of the interview transcripts using the qualitative analysis software NVivo 11 was conducted. Following the initial round of open coding, five team members completed collaborative, selective coding [25]. Themes from the analysis were developed, discussed and debated at successive weekly team meetings which shaped the analytical themes emerging from the data. These meetings also integrated insights from the surveys with those from the interviews and compared the findings from the two data sets.

### Surveys

An online survey of the HiAP policy network in the SA public sector, which conforms with Klijn and Koppenenjan’s conception of a policy ‘network of interdependent actors’ [26], was conducted during May and June in 2013 and repeated in 2015. Individual actors in the HiAP policy network were identified with the assistance of HiAP staff and comprised public servants who had had some contact with HiAP since 2007. In 2013 and 2015, the network involved 435 and 483 public servants, respectively. The survey samples for 2013 and 2015 were selected from these networks and included only people who were working within the SA Government at the time of each survey. In 2013, 373 public servants were invited via email to participate in the first survey. 168 (45%) of these people provided meaningful responses by answering survey questions beyond the initial demographic questions. Six people (2%) answered only the demographic questions and their responses were excluded from the analysis. 199 people (53%) did not respond at all. In 2015, 339 people were invited via email to participate in the second survey. 151 (45%) of these people provided meaningful responses by answering survey questions beyond the initial demographic questions. 25 people (7%) answered only the demographic questions and their responses were excluded from the analysis. 163 people (48%) did not respond at all. Each potential respondent was contacted four times [27].

Most of the questions remained consistent between the two surveys; however, additional questions were added in 2015 to capture data about changes in the HiAP approach and the surrounding political context. These changes had been identified during the preceding interviews and the documentation of developments in the log, and the 2015 survey was used as a tool to gain further quantitative insights.

The surveys elicited information about respondents’ awareness of the HiAP approach, experiences of collaborating with HiAP work, and perceived outcomes of HiAP work. An awareness of HiAP was deemed a necessary precondition for valid responses to questions about experiences of collaborating with HiAP work, therefore the analyses were limited to those respondents who indicated in the survey that they had heard about HiAP (2013, *n* = 148 (40%); 2015, *n* = 105 (31%)) and were working in a department other than the Health Department (2013, *n* = 83; 2015; *n* = 61). The majority of non-health respondents had worked in the SA Government for five years or more (2013, *n* = 50 (88%); 2015, *n* = 59 (97%)) and approximately half were executive level or senior management staff (2013, *n* = 42 (52%); 2015, *n* = 25 (41%)). Non-health respondents were from 14 and 13 different departments/agencies in the 2013 and 2015 surveys, respectively.

#### Data analysis

Numerical data from the surveys were exported from Survey Monkey directly to an SPSS file and analysed using IBM SPSS Statistics 22. To ensure the accuracy of the data file, the SPSS data file was proofread against the original data in Survey Monkey. Cross tabs were used to compare relationships between responses to selected questions, and between surveys. Data from the open ended questions were exported from Survey Monkey and analysed thematically using NVivo 11.

Interviewee and survey respondents are identified by the sector in which their employing department/agency has been categorised, for example Health Department staff are identified as from the health sector and staff from the Department of the Premier and Cabinet are identified as from the governance sector. Departmental/agency names have changed and departments have been split and merged over the timeframe of this research. Providing a more general categorisation by sector helps to identify the activity of the department/agency without having to track the continual changes in names and structures arising from departmental restructuring.

All data collection activities received prior approval from the Flinders University Ethics Committee and the SA Health Ethics Committee. Where an interviewee may be identified by their quote, their consent has been sought for its use.

## Results

Our findings first describe the range of initiatives in other sectors engaged in by HiAP, then show how non-health sectors shaped the implementation of HiAP through their ideas and institutional contexts. Finally we describe agency of the actors and their networks active in HiAP.

### HiAP action initiated in other sectors

Table 1 draws on the document analysis to show the range of actions in the other sectors related to health. The initiatives addressed a range of social determinants of health which from 2008 to 2013 were identified by the use of a Health Lens Analysis (HLA). HLA provides a rapid, highly policy relevant assessment of the impact of policies outside the health sector [28]. The Health Lens Analysis process is the practical methodology that supports the HiAP approach in SA. Its primary intent is to highlight the connections and interactions between health and the core business of other sectors [29]. It involves five stages: HiAP staff engage other departments in cross sectoral work to promote health, gathering evidence, generating outputs that will be useful in progressing a health agenda (reports, recommendations, policy), navigating those outputs through bureaucratic and political processes and structures to evoke change, and evaluation of the effectiveness of the process. From the interview data, public servants with a favourable disposition to HiAP were identified in 12 out of 15 government departments/agencies. These actors shared an appreciation of the role of the health lens projects in prompting a shift in thinking within their departments, contributing additional resources (staff and funding) and creating space for a different focus of work within their departments which would not have been pursued without collaborating with Health. For example, an interviewee explained:

I just think all of that knowledge and learning and opportunity we had that came out of HiAP is one amazing thing… and that continues on, like those people are spotted around, those projects are around, those outcomes are around (Executive/Senior management, Governance sector, 2016).

**Table 1:** Implementation of Health in All Policies in South Australia (2009 to 2016) showing sectors involved

Description of initiative	Key sectors involved	Intermediate or health outcome claimed
Parental Engagement with Literacy Health Lens Analysis (HLA)	Education	Change to Education dept. literacy and numeracy policy regarding parental engagement
Aboriginal Road Safety- Drivers Licensing HLA	Emergency services; Transport; Justice; Correctional services; Education	Minor increase in Aboriginal people with driver’s licences in remote communities, which is likely to reduce road accidents and incarceration rates.
Promoting International Students’ Health and Wellbeing HLA	Education; Multicultural	Resources for international students on health services access and maintaining well-being produced
Healthy Sustainable Regional Communities in the Upper Spencer Gulf HLA	Primary industries; Trade & economic development	Awareness of importance of considering health and equity issues in regional planning increased in trade and economic portfolios and data atlas to support this
Healthy Weight: A Desktop Analysis and Implementation Plan	Health; Planning & infrastructure; Community services & welfare; Primary industries; Environment & natural resources; Education; Correctional Services; Justice	Large range of departments made aware of the impact they have on population average weight and the potential actions they can take to achieve the healthy weight target. Progress on strategies within departments reported to Parliament annually.
Health Promoting Transit-oriented Developments (TODs) HLA	Planning & infrastructure; Transport; Urban planning & development	Contribution to development of suburbs that have lower ecological footprint and which encourage walking, cycling and use of public transport. Produced tool to assess health impacts of future TODs.
Local Government HiAP Approach: Castle Plaza Transit-orientated Development HLA	Local government	Greater awareness of health issues that may be associated with the TOD
Active Transport – Economic Assessment for Cycling and Walking and Cycling Strategy	Planning & infrastructure	Strengthening the case for better provision for cycling and walking by providing health and well-being rationale
Regional Migrant Settlement	Trade & economic development; Multicultural	Minimal impact but provided some rationale for considering health and wellbeing in migrant settlement
Alternative Water Supplies – Water Security	Environment & natural resources	Raised awareness of potential positive and negative health impacts of increasing the re-use of stormwater, greywater and rainwater during policy development process
Digital Technology: Increased Broadband Use	Education	More awareness of the importance of broadband access in terms of gaining access to social determinants including employment, education and housing and the health equity implications of some groups not gaining access.
*Provision of advice, evidence and capacity building around how the cross-sectoral 7 Cabinet Priorities can contribute to health and wellbeing:* - Every Chance for Every Child: Capacity building across Government - Safe Communities, Healthy Neighbourhoods - An affordable place for everyone to live - Realising benefits of mining boom for all - Premium food and wine from our clean environment - Growing Advanced Manufacturing - Creating a Vibrant City	Each of the 7 Strategic Cabinet Priorities were led by Ministerial Taskforces supported by Senior Officers Groups. Initially these were led by Premier and Dept. of the Premier & Cabinet (DPC), in partnership with Minister and the government department with primary responsibility for policy issue. Over time the relevant Minister and department took on primary responsibility for each of the priorities. Premier and Minister for Education supported by DPC and Education sector Premier, Commissioner of Police and Minister for Health supported by DPC, Justice, Health Premier, Treasurer supported by DPC and Finance Premier, Minister for Industry & Trade supported by DPC and Trade & economic development Premier, Minister for Primary Industries supported by DPC and Primary industries Premier, Minister for Industry & Trade, DPC, Trade & economic development Premier, Minister for Planning supported by DPC and Planning & infrastructure	Bringing an awareness of the health impact of the work of each of these taskforces and encouraging them to make health a key consideration
Premier’s Healthy Kids Menus Taskforce	Health; DPC; key stakeholders including Australian Hotels Association, Restaurant & Catering Association and Clubs SA, Heart Foundation, CSIRO and Parent representatives- chaired by the Parliamentary Secretary for Health	Recommendations for entertainment venues about how they can support healthy eating for families
90 Day Change Project – One Government: Working together for integrated policy that meets citizens’ needs	Health, DPC, Office for the Public Sector, Environment & natural resources	Lessons from the HiAP experience of cross sectoral working directly informed this initiative and underpinned the strategies developed as part of it.
Applying HiAP Principles to work with Public Health Partner Authorities (PHPA) across SA	Involves a range of Government and non-Government partners, including Environment & natural resources. Policy focus - healthy parks, healthy people Planning & infrastructure. Policy focus - planning reform, urban renewal and healthy built environment SA Council of Social Services. Policy focus - The role of non-government sector in public health planning system University of South Australia. Policy focus - research policy translation - social isolation, older people and the built environment Community services & welfare. Policy focus –whole of government Wellbeing Framework concept and measurement; increasing access to healthy nutritious food for vulnerable people at risk of hunger.	Relatively new initiative (since 2015). The legislative basis of the PHPA promises to help health become more prominent in the activities of those agencies that sign up to be a PHPA.

Comments from staff where a favourable disposition did not exist in their departments indicate that the reasons for this included that the focus of the HLA was already their core business and they could have undertaken this work themselves without the use of a HLA or involvement of HiAP:

…the approach appears to me to have been ‘Well, here’s an issue. We think this needs to be tackled. We think it needs to be done like this.’ Whereas we, having that core sort of business responsibility, have already identified that quite solidly and have, you know, work in place (Education sector, 2013).

Table 1 also shows the outcomes that were noted in the interviews to have resulted from each of the areas of cross-sector activity. In each case these outcomes were addressing a social determinant of health.

**Fig. 1:**
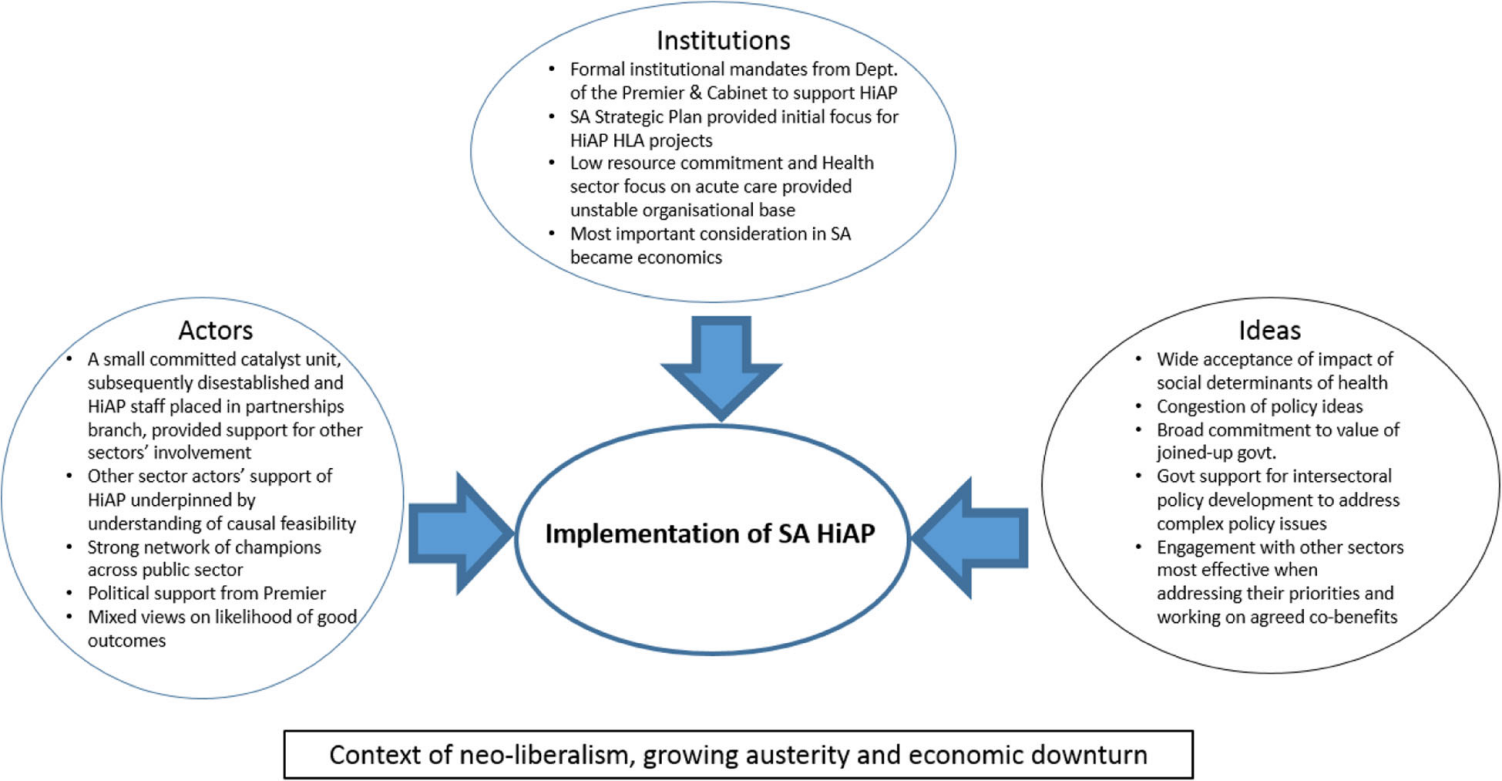
Intersection of Institutions, Ideas and Actors in HiAP in South Australia

Next we discuss the three pillars of the institutional analysis we have used to frame our analysis, namely ideas, institutions and actors. An overview of our application of this framework is provided in Fig. 1.

### Dominant policy ideas in South Australia

HiAP’s premise that health is influenced by social determinants that are largely outside the health sector, and HiAP’s intent in influencing work on these social determinants, was reasonably widely accepted among South Australian public servants. Our survey indicated that most rated their understanding of HiAP as excellent or very good/good (2013, *N* = 82, *n* = 57, 70%; 2015, *N* = 60, *n* = 39; 65%) while less than one-third lacked at least a good understanding of HiAP. Similarly less than 30% of respondents (2013, *N* = 69, *n* = 28; 2015, *N* = 44, *n* = 14) agreed or strongly agreed that the impact of their departmental policies on health were not clear. Most reported that the policies in their department did have a moderate or strong impact on health and wellbeing (2013, *N* = 73, *n* = 60, 82%; 2015, *N* = 61, *n* = 55, 90%).

A majority of respondents agreed or strongly agreed that the link between the work of their department and health outcomes was supported by evidence (2013, *N* = 68, *n* = 38 (60%); 2015, (*N* = 42, *n* = 23 (55%)), although some did indicate problems. A few noted that “health” is equated with hospitals and illness, and a community services and welfare sector director suggested the alternative term of “social well-being… you’d probably get more buy-in because public servants sort of like to think that’s what they’re about”. An education sector director noted “challenges around aligning the language and knowledge and processes of one sector” and that it takes time to “know each other’s world, developing a common language… filing it down so that conversations are more and more specific”. Despite these cultural difficulties other sectors noted that linking their agendas to health could have benefits. A coordinator in the environment and natural resources sector saw that conserving the environment is “perceived to be something outside… so linking our work to health, linking our work to tourism and fun and wellbeing… not only does it give conservation more impetus but it actually makes us part of something bigger”. Overall, about half of respondents felt that collaborations with HiAP were very or somewhat effective in achieving the goals of their department/agency’s core business (2013, *N* = 73, *n* = 37 (51%); 2015, *N* = 52, *n* = 30 (58%)). Thus a clear majority of the public servants were supportive of the ideas behind HiAP.

#### Congested policy environment

As in most modern liberal democratic capitalist states, the policy space in South Australia is congested. Our analysis, however, highlights that there was a degree of coherence between the ideas underpinning HiAP and broader initiatives designed to reduce barriers between government departments and encourage actions across bureaucratic barriers. The South Australian Strategic Plan (SASP) set goals for health and well-being and action on a range of social determinants. Until 2013 all Chief Executives of government departments were accountable for the achievement of these goals. A political advisor from the governance sector noted the link between the SASP and HiAP:

In the 30 Year Plan [for Greater Adelaide] and the South Australian Strategic Plan, HiAP provided the opportunity to ask for each of the general policy propositions ‘what’s the overall health impact of this?’ which would not necessarily be asked otherwise. Whereas traditionally the questions have been economic impact, then environmental impact and sometimes social impact, but health impact has not been a traditional question in policy development. (Political staff, governance sector, 2013).

HiAP was one of many strategies, new ideas and policy directions in the State and so other sectors had to be convinced of its utility and fit with other intersectoral government priorities. Our analysis showed how HiAP was implemented on shifting policy sands as part of a changing and fast-moving agenda of government, as summarised by a senior executive:

So what Health in All Policies has had to do is had to adapt to something in South Australia which has been quite difficult because we’ve still got the South Australian Strategic Plan but layered on top we’ve got the seven strategic priorities that they had to adapt and fit into. Then since the election we’ve got also another ten economic priorities and extra things happening… it’s sort of the principle of being at the centre of other people’s problems, like how do you help solve their problem with them and how can HiAP help, is hard when the landscape is changing so quickly. (Executive/Senior management, governance sector, 2014).

Similarly, a senior executive in the governance sector noted that “shiny things” like HiAP had to play a delicate game of “jumping” on to “the new shiny thing” but also remaining visible in their own right. Within the context of leadership changes within the Government, a project like HiAP was at risk of being seen “as the last person’s idea so they won’t touch it”. Maintaining relevance with other sectors meant HiAP had to traverse the unpredictable and fast moving policy terrain. From 2012, with a change of Premier, new “Strategic Priorities” were mandated and these became the business of multiple departments and appeared to assume priority over the SASP. Thus departments had a common purpose which was frequently noted in the interviews as essential for effective action. A senior officer from the governance sector (2013) noted that HiAP had “got other agencies across government working with them and thinking about health in a different way and health isn’t just Health’s business, it’s everyone’s business”.

#### Commitment to joined-up government

Both South Australian Premiers in office during our study period had a strong commitment to reduce the silos in government and encouraged joined-up approaches.

…what I had seen before was always this incredible frustration of things being done in silos and being treated as symptoms… This was to get them from thinking narrow ‘this is my empire’ to thinking about ‘this is – we’re all in it together’. (Former Premier, June 2013).

In the words of a public servant, this strong political support gave a “strong philosophical agreement of the need to work across sectors, settings and disciplines to improve outcomes”, a sentiment that was echoed by many of those surveyed and interviewed. It also meant HiAP was one of many cross sectoral initiatives, including a Cabinet-initiated 90 day change project very similar to, and in part, modelled on the HiAP health lens. Such new initiatives required HiAP to jostle for policy space in the other sectors but as a senior executive in the trade and economic development sector (2015) noted, “there’s a ripple in the pond that’s coming out of there of which is a tailwind for Health in All Policies”. HiAP was seen as a good model for collaborative work to further the Government’s agenda of joined-up government:

Health in All Policies provides a potential template for engaging in other policy development processes, where the aim is to deliver an outcome for the state from a whole of government perspective, rather than the perspective of a single department. (Senior Executive, finance sector, 2013 survey).

The importance of HiAP in assisting other departments to focus on health was well-illustrated by the example of urban planning:

When we do our planning work we have to look at transport issues, environmental issues, social equity issues and a whole range of other things and we try and merge those all together and balance them in one way or another. Until relatively recently the health elements, which oddly enough was one of the great reasons why planning was born, but the health elements had slipped off the agenda and what the Health in All initiative did was actually re-establish its place within a land use planning framework that could then enable us to craft and re-craft policy in different areas of our work, so in that sense for us it’s been a wildly successful initiative. (Senior Executive, planning and infrastructure sector, 2013).

### Institutional context

SA HiAP was implemented by a State Government within a federated system in which neo-liberalism dominated public policies, including those of the South Australian Labor Government [30]. Neo-liberalism has been defined by Harvey [31], p.2 as a hegemonic discourse that proposes that human well-being “can best be advanced by liberating individual entrepreneurial freedoms and skills within an institutional framework characterised by strong private property rights, free markets and free trade”. Neo-liberal policies have been manifest in Australia through public spending cuts, privatised public services, and adoption of private sector modes of operation [32]. While the hegemony of neo-liberal ideas meant the policy space for the advancement of social and health innovation was limited, there was some political commitment to do so despite the intensification of an austerity agenda over the five years of the study.

#### HiAP in an institutional environment dominated by economic considerations

Respondents were clear that the economy was the top concern for policy makers. One director argued that aligning the separate goals of successful regional mining and developing a healthy community was only possible if both goals assisted economic development. A transport executive noted that while they co-operated with HiAP there would be “lots of resistance” to using the health lens on “very major projects” such as a major new expressway or additional car parking near the city. Other executives in non-health sectors noted the economic argument that investment in health through their sectors’ activities would save money in the future. An urban planning and development sector executive in 2013 spoke of the need to work towards a “pre-emptive health approach as opposed to a reactionary health approach, if we’re not supporting that in what we do then we’re producing communities that will be unhealthy in the future so we need to future proof”.

When HiAP was initiated in 2007 SA’s economy was buoyant and anticipating a mining boom, which never eventuated. Therefore, from 2013 the policy agenda shifted as the economy experienced shocks as its manufacturing base contracted, including the loss of 3000 automotive industry jobs. Following this, the State Government became even more concerned with economic goals, sidelining the SASP and seven strategic priorities and replacing them with ten economic priorities that emphasised job creation to reduce increasing unemployment. Comments from 2015 interviews reflect this growing institutional concern with the economy and job creation. Repeatedly the respondents noted that the government was focused on “jobs and economic development and diversification of the economy” (Governance sector, 2015) with the effect that it:

...then creates - anything that takes away straight commercial outcomes or economic outcomes means that the long term benefits of things like health, or even to the extent of, you know, what is sensitive urban design, etcetera, starts to not become high on the priority for what people are trying to achieve. (Urban planning and development sector, 2015).

The intensification of economic dominance in policy led to some fearing that there is little space for long term social objectives. A senior executive from the management, environment and natural resources sector in 2016 noted that the declining economy had created a crisis mentality which led to less incentives for other sectors to cooperate with HiAP. Others expressed similar views:

I think there is significant pressure on our department to find millions of dollars of savings and effectively the response [to implementation of HiAP recommendations was] ‘We’re not doing it’ whereas before, you know, I think there was a real cooperative sort of approach. (Education sector, 2013).

…so far so good, but in general when you get significant budget cuts it does tend to, as I said, pull your work right back to really what’s the core interest. (Environment and natural resources sector, 2014).

Health policy actors knew the cuts in their sector, especially those to health promotion, posed a risk to the commitment from other sectors. One senior executive noted:

[this] is a huge risk to the HiAP process because nothing starts a stampede more than the first animal running and then everybody else runs away so there’s a risk around that… and the other reality is that we might find less willing partners at the moment because we aren’t able to offer as much as a health system in terms of our own resource. (Health sector, 2013).

These fears resonated with comments such as this one questioning the health sector’s own commitment to health promotion:

We currently have a health system that has had health promotion and preventive health programs ripped out of it. (Community welfare, 2013 survey).

In this adverse environment for prevention and promotion in the health sector it appears that the support for HiAP from powerful public servants in other sectors was able to keep the policy space open for HiAP. The support for HiAP in SA in the health and other sectors was also reinforced by the international links it had established with the World Health Organization (WHO). Staff from the HiAP Unit were involved in WHO training and resource development. A high profile joint conference was held in March 2017. This international profile appears to have acted as a bulwark against the pressures of austerity. Additionally, joined-up government - of which HiAP was viewed as a positive example - was seen as a strategy to deal with austerity and in 2013 the Premier justified the strong commitment to joined-up government in terms of its economic benefits, making HiAP politically palatable:

so in a sense what we were trying to do in a range of other areas, like with homelessness being joined-up responses, or joined-up responses in dealing with kids who are dropping out of school... here’s an area where in fact investment in health is actually about a better economy as well as a better society, more productivity. (Former Premier, 2013).

#### Formal mandates for HiAP

The Executive Committee of Cabinet Chief Executives’ Group provided an important formal mandate because it contributed to the institutional framework and the governance and authorising structure for HiAP from 2008 to the CEs’ Group’s dissolution in 2012. Subsequently, from 2012 to 2014, such oversight and guidance was provided to HiAP by seven Senior Officers’ Groups which were each responsible for the implementation of one of the Cabinet priorities (see Table 1). An important theme from the survey and interviews was that HiAP would benefit from having a more formal institutional structure and so becoming a routine part of government business, as a senior executive noted:

Currently based on personal relationships between officers, not part of the ‘system’. (Environment and natural resources sector, 2013 survey).

The other sector interviewees who were most committed to the approach argued strongly that it needed to be embedded as a systemic model across the public sector. A director argued that processes of broader public sector reform had to be supportive:

I think it was really too much to ask for them to have actually left a systemic change in the system. I think that the systemic change there is a broader challenge for public sector reform and looking at the Office of Public Sector Management… but I’m not witnessing - I wouldn’t say that I’m witnessing high levels of collaboration, at a systemic level anyway… so I think the collaboration stuff is a fairly isolated - it’s on an isolated basis rather than a generalised one would be my view at this stage. (Senior executive, trade and economic development sector, 2015).

Institutionally HiAP in South Australia was a primarily horizontal activity between state government departments and did not involve federal agencies (except through brief informal consultations about how State Government HiAP projects may affect work being undertaken federally). HiAP initially only had limited local government involvement. From 2015 engagement with local government was supported by a new Public Health Act (the *South Australian Public Health Act 2011*). The Act required the health minister to provide health advice to other government departments; local councils to develop regional public health plans; and the health minister to establish Public Health Partner Authorities (which could be other government departments) [33]. The importance of this legislation in linking in other sectors and establishing public health planning processes across State and local government was noted by many respondents, for example:

Well [the impact of the Public Health Act] is still probably emerging but I think that whereas in the past Health in All Policies was a primary driver of the way in which Health was embedded across other departments’ policy agendas, I think now the Public Health Act is pretty much the main driver and I think Health in All Policies is having to kind of come in behind that and ensure that what it does is consistent with the intent of the legislation. I think it’s more about Health in All Policies adjusting to support the implementation of the legislation rather than Health in All Policies being a separate process in itself. (Governance sector, 2014).

By late 2016, four non-health State Government agencies had been formally declared as Public Health Partner Authorities. A health sector executive explained that this would provide an opportunity to make HiAP “part of everyday business” through formal partnerships. While HiAP is not explicitly referred to within the Public Health Act, the Act does provide a regulatory mechanism to support HiAP in its quest to work with other sectors.

#### Resources and management support

Monetary, human and other resources are important in driving the performance of public sector institutions and actors and are vital aspects of the institutional framework for HiAP.

A small catalyst unit was established in the Health Department to implement the HiAP approach (at its peak comprising six staff). This unit was perceived to offer a dedicated pool of skilled staff that could provide assistance across Government, and was largely responsible for creating and maintaining a networked, horizontal governance for HiAP across many State Government departments as well as a connection to powerful decision-makers through its mandate from the Department of the Premier and Cabinet. The value of this unit to policy players in other sectors was well-recognised by respondents:

if Health in All Policies didn’t exist as an agency with the resources that are behind it, I think that there would be a risk that it would fall over. (Governance sector, 2013).

I think one of the very real strengths is the HiAP team’s expertise… in getting high level access into other departments and having a strong imprimatur. (Health sector, 2013).

The perception of commitment and visibility of HiAP core staff was noted:

…they’re always there championing Health in All Policies. They’re quite – they’re out and about so they are, to me they are highly visible in what they do and how they do that, not only in Health but across government. (Health sector, 2013).

…where Health in All Policies worked really well over the last, say, three to four years was that there was a dedicated unit and they drove it a lot. They got the people around, government around the table and supported implementation; they did project management to support the Health in All Policies programs. (Governance sector, 2014).

The willingness of other sectors to commit resources to HiAP activities was an important indicator of support for the initiative and significant in-kind (staffing) and financial contributions were made by partner agencies to HiAP projects. Approximately half of survey respondents in 2013 either strongly agreed or agreed that collaborations with HiAP create additional work (2013, *N* = 74, *n* = 39 (53%)); by 2015, this had reduced (*N* = 44, *n* = 17 (39%)) probably reflecting the reduced project activity of the HiAP staff. However, most respondents disagreed or strongly disagreed that little benefit is gained from the extra work (2013, *n* = 20 (51%); 2015, *n* = 12 (71%)), indicating a supportive view.

Conversely, concerns were raised consistently during interviews between 2013 and 2015 about the sustainability of a dedicated HiAP Unit:

the HIAP Unit has the skills and the effort and dedicated resources… but it’s probably an unsustainable model. It needs to bubble out, out of the Unit and be the way that Health does business - or indeed how anybody in government does business… so it’s vulnerable but also it’ll only ever have limited impacts, so there’s four in a team or how many there is in the team, and they’ve got so much budget. It will – unless it leaves a legacy where people operate differently then – it will only ever be what it is and only have a certain amount of impact, which is probably different from being sustainable, but it’s only ever going to be limited. (Primary industries sector, 2013).

Most of the HiAP Unit’s work was project based and thus opportunistic rather than system-wide. In 2014 the unit was disbanded and staff working on HiAP initiatives had, in their words “to work below the radar” because the Health Department entered a period of severe budget reduction and the entire initiative was felt to be at risk. The HiAP staff were restructured into a Strategic Partnerships Team in a new Public Health Partnership Branch from where the HiAP work continued as an approach to working intersectorally, although with less visibility than previously. The HiAP work was protected by a renewed Memorandum of Understanding with the Department of the Premier and Cabinet - *Systematising Health in All Policies*, signed in early 2014. The skills that were reported by the staff dedicated to HiAP and observed by those in other sectors which were important to working with other sectors are summarised in Table 2.

**Table 2:** Skills used by HiAP staff to encourage the involvement of other sectors

Strategic	
• Establishing a broad shared strategic vision with other sectors and then determining how to achieve that in practice	
• Managing up in health sector and across to Department of the Premier and Cabinet to ensure support for HiAP work, and building other external alliances	
• Taking a helicopter overview of the entire initiative	
• Monitoring the ways in which the public sector environment is changing and adapting to survive	
• Watching for windows of opportunity to progress HiAP work and navigate recommendations through decision making hierarchies at times when success is most likely	
Knowledge	
• Working to understand the core agenda of other sectors	
• Detailed understanding of SDH and how the core business of other sectors may influence population health	
• Ability to interpret evidence and translate it in a way that is relevant to the core business of other sectors (e.g. evidence on link between literacy and health or evidence on links between urban planning, walkability and the creation of health promoting spaces)	
Relational	
• Being proactive and making cold calls to public servants in other sectors	
• Building and fostering a broad and supportive network in public service for HiAP	
• Relationship building which includes confidence with networking and making informal contact with actors from other sectors (eg coffee and discussions)	
• Shepherding the on-going HiAP work from behind the flock by nurturing collaborations	
• Confidence working at the boundary and linking organisations across different sectors	
• Confidence to put the business of other sectors first, and to work with them to identify co-benefits to advance the other sector’s priorities while also addressing Health’s priorities	

Institutionally the strongest support for HiAP came from the structures established with the Department of the Premier and Cabinet. This central government support was vital to the continued commitment of other sectors and was more enduring than from the Health Department where a new minister, driven by extreme budget pressures, dramatically reduced support for health promotion [34]. The most promising institutional development was the new Public Health Act.

### Actors

Staff within the HiAP Unit in the Health Department were crucial actors in winning over other sectors to HiAP. They strove to avoid what they referred to as “health imperialism” and sought win-win solutions to policy problems. They worked to understand other sectors’ agendas so the resulting policies offered benefits both for health and the particular sectoral agenda (see Table 1).

An important reason for the commitment from actors from other sectors was that they appreciated the causal feasibility of the HiAP work. This appreciation reflected the work of the HiAP Unit through Health Lens Analyses exercises designed to link the activities of other sectors to health outcomes. In the interviews and surveys public servants gave examples of links between their work and health outcomes in relation to literacy; urban design to encourage active lifestyles and low carbon behaviours; increasing training so more people have jobs; and creating healthy communities so that they are attractive for economic investment. A senior education executive commenting on the factors that promote collaboration between their department and HiAP wrote:

Alignment of strategic objectives and the commonality of social factors that influence both education and health outcomes (2013 survey).

A further factor that influenced the extent of actor support was whether HiAP was seen to be likely to achieve outcomes. About half of the survey respondents agreed or strongly agreed that the outcomes of collaborations with HiAP were unclear (2013, *N* = 68, *n* = 35, (51%); 2015, *N* = 43, *n* = 20 (47%)) and some of the interviewees questioned the extent to which outcomes were achieved. For example an executive noted that HiAP had “given us another window;” then queried “has that generated outcomes massively different to what we’ve already got?” and concluded HiAP hadn’t. Such perceptions are likely to undermine support from other sectors.

Exworthy, and de Leeuw and Peters have argued that a potential threat to HiAP’s technical feasibility is that policy actors would see the initiative as too complex [16, 35]. However, few respondents agreed or strongly agreed that collaborations with HiAP were more complex than other areas of their work (2013, *N* = 72, *n* = 10 (14%); 2015, *N* = 50, *n* = 8 (16%)). As one executive/senior management survey respondent commented, “there are many complexities in our work. HiAP is but one factor to consider” (Community services and welfare sector, 2013 survey). Our interviews suggested that because public servants are used to dealing with both complicated and complex problems, any perceived complexity of HiAP is not a barrier to its feasibility. A policy officer said HiAP was:

No more or less complex than any other. Simple idea, just a way of doing good policy… So the topics are complex but the broad aim of HiAP in terms of bringing people together to consider the health outcomes of policy is not that complex. (Governance sector, 2013).

A senior executive in the Department of the Premier and Cabinet echoed these comments: “I don’t think its complex at all; I just think its common sense.” (Governance sector, 2013).

### Networks and champions

HiAP had high level political support especially from the first of the two Premiers under which it operated. It got on to the crowded policy agenda partly because of and through a Thinkers in Residence Program, championed by the State Premier at the time who wanted to build joined-up government [36]. The Premier’s support was backed by strong support from senior executives in the Department of the Premier and Cabinet (DPC), which was vital as an authorising environment for public servants, as shown by a senior executive who noted:

DPC’s a lead agency [for] Health in All Policies, so from pretty early days I’ve been one of the people driving the work within the South Australian government. There’s been lots of governance changes now and my job’s different now but I’ve continued that sort of role of being one of the central drivers and a central contact for Health in All Policies and other intersectoral sort of challenges basically. (Governance sector, 2013).

Political support from Chief Executives of agencies was also reported as important and the interview data suggest (unsurprisingly) that where the support was strong public servants found it easier to co-operate with HiAP. Sometimes public servants noted the need to win this support. Around one third of the respondents in the 2013 survey (*N* = 68, *n* = 28 (41%)) and a quarter of respondents in the 2015 survey (*N* = 43, *n* = 11 (26%)) strongly agreed or agreed that they had concerns about how to justify collaborations with HiAP to their senior management. One non-health senior executive summed this up saying “you’ve got to win the hearts and minds of the people that lead the organisation” (Education sector, 2013). In a similar vein a health sector officer noted the importance of senior engagement from other sectors:

…I mean the fact that we have had other Chief Executives writing to our Chief Executive to talk about the importance and value of the work, I think those sorts of elements have been critical. (Health sector, 2014).

While the support from Chief Executives varied, we found evidence that a number were supportive. One noted that they had been able to continue their support as they moved from role to role:

...but I think because I’ve had the interest in Health in All Policies and worked around it in other places it’s been like a sort of a natural progression for me to jump in and do some leadership. (Executive/senior management, environment and natural resources sector, 2016).

This officer continued to explain the way in which champions had been created from the perceived success of HiAP and were now “spotted around the public sector” as they moved into other senior positions of influence, taking with them their support for and understanding of HiAP as an effective intersectoral policy approach that achieves multiple outcomes.

One result of the relative longevity of HiAP was the development of an on-going supportive network of middle to senior public servants who are broadly supportive of HiAP. However, the effectiveness of this network and the application of the HiAP approach was threatened by the public sector cuts described earlier. The HiAP Unit was disbanded, staff moved to the new Public Health Partnerships Branch and formal engagement with other sectors was largely put on hold from the end of 2013 until early 2015, and projects were temporarily delayed. The following respondent from Health highlights the consciousness within HiAP of the importance of these network relationships:

I think that the current members in the Strategic Partnerships Team, because they are the previous members of the Health in All Policies Unit, are trying their damnedest to, under the radar, maintain those relationships as best as possible because the cycle will happen again and I think the most important thing to protect are those relationships that were forged in the last four years, four to five years, because if they are affected then we’ll never ever get off the ground again, ever. (Health sector, 2014).

A non-health sector respondent explained the impact of the retreat from positive action to working defensively and “under the radar” for their HiAP project as being difficulty getting joint sign off from their Chief Executive and the Health Chief Executive. While the respondent was able to get the signature from their CE they reported that “I notified Health in All Policies people that that had happened so that they could prepare the letters and get them signed by their Chief Executive and all fell into a big black hole… I did follow up with them just to see what was happening and I think they’d been under lots of pressure and hadn’t done them”. The HiAP team’s perceived need to “go under the radar” was clearly frustrating to other actors and is likely to have weakened support for HiAP. However there is evidence that experience with HiAP had encouraged actors in other sectors to consider health implications independently of their involvement with HiAP as shown by an actor from the planning and infrastructure sector discussing how they took health into account while drafting and implementing new planning legislation, and how they were pleased to see “that there’s a degree of buy-in now from other parts of the agency, thinking about the implementation and therefore thinking about how they plug into health-related issues.”

## Discussion

Drawing on the framework offered by Howlett, Ramesh and Perl we have analysed how the institutions, ideas and actors shaped the policy sub-system in which HiAP operates and the many features of these systems which supported constrained actors in other sectors considering health [18]. Here we identify the key factors that encouraged non-health public servants to advance the HiAP agenda. They are summarised in Table 3.

**Table 3:** Key factors encouraging non-health sector commitment to Health in All Policies

- Supportive bureaucratic HiAP policy network	
- Political mandate	
- Move from project focus to institutionalisation and systematisation	
- Finding a fit between HiAP ideas and the dominant economic paradigm	

### Importance of a supportive bureaucratic HiAP policy network

We found that HiAP in South Australia was supported by a network of public servants who understood the social determinants of health and saw how their sectors contributed to health. Exworthy notes that such networks can be a powerful way to bring about action on social determinants [16]. Such networks have been observed in other jurisdictions implementing HiAP [37, 38]. An important part of the network formation and maintenance outside health appeared to be the policy entrepreneurs who support HiAP. Kingdon saw such actors as those who invest their own resources in promoting a policy idea [39]. In South Australia these were found at senior levels of government including in the Department of the Premier and Cabinet, in the HiAP Unit and throughout many government departments. A senior executive in the Department of the Premier and Cabinet gave the initiative considerable legitimacy across the government sector. The entrepreneurs within Health were careful to work closely with other sectors in a way that met the other sectors’ agendas, but their position was marginalised within the Health Department which increasingly took a more hospital focus. HiAP importantly gained key support from senior and middle level staff in a range of sectors. These staff either saw the logic of their work promoting wellbeing or saw HiAP as a means of demonstrating their commitment to the broader government agenda of breaking down silos within the public sector. HiAP in South Australia had more senior policy entrepreneurs outside Health than it did within Health which increased its credibility with other sectors. We also noticed that as the non-health actors moved into new positions they often took their knowledge and enthusiasm for HiAP with them. The evolution of the supportive policy network in South Australia is reminiscent of the type of Advocacy Coalition that emphasizes the importance of coalition formation to support new policy [40, 41]. Coalitions are groups of actors that share belief systems, coordinate actions around these beliefs and encourage members with common beliefs to coalesce around a policy issue [42, 43]. This type of coalition had certainly evolved in South Australia and has contributed to the continuation of HiAP despite the state context becoming more focused on economic rather than health and social goals.

### Political mandate for other sectors’ involvement

Bridging the nexus between research, policy and practice is a political process which requires a sophisticated understanding of governance networks and institutional arrangements [44, 45]. Political support for cross government action was vital to winning the commitment of other sectors in South Australia. The Premier in office when HiAP started was supportive because it fitted with his joined-up government agenda. His ambitions to achieve this agenda were strongly supported by a “Thinkers in Residence” program in which a number of Thinkers argued for the joined-up agenda and one specifically for HiAP [36]. Both Premiers in government during the period of this study made strong public statements about working across government, and initiated policy initiatives that sat alongside HiAP to encourage action across sectors. Without their strong political leadership for the concept of joined-up government the HiAP initiative would not have had as much legitimacy with other sectors. The HiAP health lens informed the new 90 day change projects which required public servants from a range of sectors and other non-government stakeholders to take a policy issue (such as barriers for low income households to the private rental market, or breaking the cycle of young offending) and devise solutions requiring cross government and intersectoral collaboration.

The initial health minister supported HiAP, and while his successor continued some support, overall, political support for health promotion, particularly within the health sector itself, was withdrawn [34]. The waning political support had the potential to compromise support for HiAP within the Government more broadly. Some other sectors were reluctant to continue support when policy entrepreneurs from the health sector were not active in promoting HiAP and when they perceived that desired outcomes would not result because health sector actors were constrained in their ability to act. It was clear that actors from all other sectors noticed the withdrawal of political support for health promotion and this made some question their continued support for HiAP. However, in general, we found that HiAP’s longevity, and the relationships that had been formed already, provided the basis for continued support from most other sectors of the SA Government, including a renewed commitment from the Department of the Premier and Cabinet. These findings indicate that ensuring other sectors’ buy-in and commitment requires a health sector which expands rather than contracts HiAP activity and establishes HiAP support staff as permanent features of the Health Department so that other sectors can rely on their presence, support, encouragement and mandate.

### Moving beyond a project basis to institutionalisation

Some actors from the other sectors perceived HiAP as being a project-based initiative that brought some advantages to their sector but which remained marginal. Institutionally, HiAP gained traction because of its appeal as a cross-government initiative. We found that within a mobile public service, where public servants regularly assumed new roles in the same or different sectors, they reported taking the skills they had learned from HiAP with them. In some situations this led to new opportunities for HiAP, such as forming partnerships with a range of sectors as Public Health Partner Authorities. The HiAP staff played a central role in establishing Public Health Partner Authorities under the new Public Health Act and they drew on existing relationships to do so, as well as seeking contact with new actors. This measure may, over the longer term lead to institutionalisation of HiAP principles [46, 47].

### Finding a fit between HiAP ideas and the dominant economic paradigm

The data that we found confirming that economic considerations are paramount in policy decision making suggested that health was valued instrumentally rather than intrinsically for the role it could play in supporting economic development. While this supported the implementation of HiAP, it also restricted its scope. In our interviews it was very rare for any policy actors to speak of the primacy of health equity or to use health as a human rights argument for their work, supporting the Government’s neo-liberal perspective in a number of ways [48, 49]. The political message in South Australia was that the public sector cuts are unavoidable but if public servants could work smarter and in a more co-ordinated way then the cuts could be accommodated. No public servants questioned the necessity for the cuts, suggestive of the observation that ‘neoliberalism has the effect of limiting what is sayable, doable and even thinkable’ [50].

The central idea of HiAP is that government intervention is essential to promote the health and wellbeing of the population. Our study suggests that when governments replace this with a focus on economic matters, public servants are unable to resist the dominant economic paradigm.

## Conclusions

Nutbeam noted that policy implementation in health promotion is most likely when there is a synthesis of plausible evidence, political vision and practical strategies [51]. Our case study of HiAP demonstrates that despite institutional constraints and shifting political support for the value of prevention and health promotion, HiAP in South Australia gained traction in other sectors. The experiences of cross-government work inspired some powerful bureaucratic actors, and HiAP initiatives led to other sectors considering health more systematically in their core activities. However other sector actors did not view HiAP as either institutionalised or the ideas behind it sufficiently supported by Government to the extent that they felt mandated to incorporate its principles in their work. Where actors had a pre-existing disposition they were keen to do so and the HiAP Unit’s work was able to support these actors.

Our conclusion is that a more systematic and mandated response to promoting health in other sectors is required. In South Australia amending the Public Health Act to provide regulated processes and procedures would ensure health considerations are built into policy development from the outset. Other jurisdictions would need to find context appropriate mechanisms to achieve this. In South Australia’s case the amendment to the Act would build on existing functions of the health minister under the Act to provide advice to the Government about health protection and promotion and on policies or measures that significantly impact on public health. Such institutionalisation of HiAP and the ideas behind it would strengthen the arm of those actors already committed and place pressure on those with less commitment. It would also protect HiAP activity from the vagaries of changing ministers with differing understandings of the importance of health promotion and disease prevention and from governments that focus on the economy to the detriment of health and social goals.

The presence of key factors such as the existence of a supportive, knowledgeable policy network, political support, institutionalisation of the ideas and approach, and balancing of the political and social goals of government are important to support the engagement of other sectors with a HiAP approach. Identifying strategies to institutionalise HiAP to ensure it becomes a normal part of intersectoral public policy development is critical to the sustainability and impact of HiAP, and vital in the face of a strong and prevalent neo-liberal economic agenda from governments.

## Abbreviations

CEs’ Group: Chief Executives’ Group
DPC: Department of the Premier and Cabinet
HiAP: Health in All Policies
HLA: Health Lens Analysis
SA: South Australian
SASP: South Australian Strategic Plan
WHO: World Health Organization
