# Correction to: Ideas, actors and institutions: lessons from South Australian Health in All Policies on what encourages other sectors’ involvement

**DOI:** 10.1186/s12889-017-4872-9

**Published:** 2017-11-08

**Authors:** Fran Baum, Toni Delany-Crowe, Colin MacDougall, Angela Lawless, Helen van Eyk, Carmel Williams

**Affiliations:** 1 Southgate Institute for Health, Society and Equity, Flinders University, Adelaide, Australia; 2 College of Medicine and Public Health, Flinders University, Adelaide, Australia; 3 College of Nursing and Health Sciences, Flinders University, Adelaide, Australia; 4 Health Determinants and Policy, Department for Health and Ageing, Adelaide, Australia

## Correction

After publication of the article [1], it has been brought to our attention that Table 1 has been formatted poorly in the original version so that the columns are not aligned with their corresponding information. The correct version of the table is presented below. The original version of the article has now been revised.

**Table 1:** Implementation of Health in All Policies in South Australia (2009 to 2016) showing sectors involved

Description of initiative	Key sectors involved	Intermediate or health outcome claimed
Parental Engagement with Literacy Health Lens Analysis (HLA)	Education	Change to Education dept. literacy and numeracy policy regarding parental engagement
Aboriginal Road Safety- Drivers Licensing HLA	Emergency services; Transport; Justice; Correctional services; Education	Minor increase in Aboriginal people with driver’s licences in remote communities, which is likely to reduce road accidents and incarceration rates
Promoting International Students’ Health and Wellbeing HLA	Education; Multicultural	Resources for international students on health services access and maintaining well-being produced
Healthy Sustainable Regional Communities in the Upper Spencer Gulf HLA	Primary industries; Trade & economic development	Awareness of importance of considering health and equity issues in regional planning increased in trade and economic portfolios and data atlas to support this
Healthy Weight: A Desktop Analysis and Implementation Plan	Health; Planning & infrastructure; Community services & welfare; Primary industries; Environment & natural resources; Education; Correctional Services; Justice	Large range of departments made aware of the impact they have on population average weight and the potential actions they can take to achieve the healthy weight target. Progress on strategies within departments reported to Parliament annually
Health Promoting Transit-oriented Developments (TODs) HLA	Planning & infrastructure; Transport; Urban planning & development	Contribution to development of suburbs that have lower ecological footprint and which encourage walking, cycling and use of public transport. Produced tool to assess health impacts of future TODs
Local Government HiAP Approach: Castle Plaza Transit-orientated Development HLA	Local government	Greater awareness of health issues that may be associated with the TOD
Active Transport – Economic Assessment for Cycling and Walking and Cycling Strategy	Planning & infrastructure	Strengthening the case for better provision for cycling and walking by providing health and well-being rationale
Regional Migrant Settlement	Trade & economic development; Multicultural	Minimal impact but provided some rationale for considering health and wellbeing in migrant settlement
Alternative Water Supplies – Water Security	Environment & natural resources	Raised awareness of potential positive and negative health impacts of increasing the re-use of stormwater, greywater and rainwater during policy development process
Digital Technology: Increased Broadband Use	Education	More awareness of the importance of broadband access in terms of gaining access to social determinants including employment, education and housing and the health equity implications of some groups not gaining access
Provision of advice, evidence and capacity building around how the cross-sectoral 7 Cabinet Priorities can contribute to health and wellbeing	Each of the 7 Strategic Cabinet Priorities were led by Ministerial Taskforces supported by Senior Officers Groups. Initially these were led by Premier and Dept. of the Premier & Cabinet (DPC), in partnership with Minister and the government department with primary responsibility for policy issue. Over time the relevant Minister and department took on primary responsibility for each of the priorities: > Every Chance for Every Child: Capacity building across Government – Premier and Minister for Education supported by DPC and Education sector. > Safe Communities, Healthy Neighbourhoods – Premier, Commissioner of Police and Minister for Health supported by DPC, Justice, Health. > An affordable place for everyone to live – Premier, Treasurer supported by DPC and Finance. > Realising benefits of mining boom for all – Premier, Minister for Industry & Trade supported by DPC and Trade & economic development. > Premium food and wine from our clean environment – Premier, Minister for Primary Industries supported by DPC and Primary industries. > Growing Advanced Manufacturing – Premier, Minister for Industry & Trade, DPC, Trade & economic development > Creating a Vibrant City – Premier, Minister for Planning supported by DPC and Planning & infrastructure.	Bringing an awareness of the health impact of the work of each of these taskforces and encouraging them to make health a key consideration
Premier’s Healthy Kids Menus Taskforce	Health; DPC; key stakeholders including Australian Hotels Association, Restaurant & Catering Association and Clubs SA, Heart Foundation, CSIRO and Parent representatives- chaired by the Parliamentary Secretary for Health	Recommendations for entertainment venues about how they can support healthy eating for families
90 Day Change Project – One Government: Working together for integrated policy that meets citizens’ needs	Health, DPC, Office for the Public Sector, Environment & natural resources	Lessons from the HiAP experience of cross sectoral working directly informed this initiative and underpinned the strategies developed as part of it
Applying HiAP Principles to work with Public Health Partner Authorities (PHPA) across SA	Involves a range of Government and non-Government partners, including: - Environment & natural resources. Policy focus is on healthy parks, healthy people - Planning & infrastructure. Policy focus is on planning reform, urban renewal and healthy built environment - SA Council of Social Services. Policy focus is on the role of non-government sector in public health planning system - University of South Australia. Policy focus is on research policy translation, social isolation, older people and the built environment - Community services & welfare. Policy focus is on whole of government Wellbeing Framework concept and measurement; increasing access to healthy nutritious food for vulnerable people at risk of hunger.	Relatively new initiative (since 2015). The legislative basis of the PHPA promise to help health become more prominent in the activities of those agencies that sign up to be a PHPA
